# Life-threatening massive pulmonary embolism rescued by venoarterial-extracorporeal membrane oxygenation

**DOI:** 10.1186/s13054-017-1655-8

**Published:** 2017-03-28

**Authors:** Fillipo Corsi, Guillaume Lebreton, Nicolas Bréchot, Guillaume Hekimian, Ania Nieszkowska, Jean-Louis Trouillet, Charles-Edouard Luyt, Pascal Leprince, Jean Chastre, Alain Combes, Matthieu Schmidt

**Affiliations:** 1 Dipartimento di Anestesia e Rianimazione, Policlinico Universitario A. Gemelli, Università Cattolica Del Sacro Cuore, Rome, Italy; 2 Medical Intensive Care Unit, iCAN, Institute of Cardiometabolism and Nutrition, Hôpital de la Pitié–Salpêtrière, Assistance Publique–Hôpitaux de Paris, Université Pierre-et-Marie-Curie, Paris 6, 47, bd de l’Hôpital, 75651 Paris Cedex 13, France; 3 Cardiac Surgery Department, iCAN, Institute of Cardiometabolism and Nutrition, Hôpital de la Pitié–Salpêtrière, Assistance Publique–Hôpitaux de Paris, Université Pierre-et-Marie-Curie, Paris 6, 47, bd de l’Hôpital, 75651 Paris Cedex 13, France; 4 Service de Réanimation Médicale, iCAN, Institute of Cardiometabolism and Nutrition, Hôpital de la Pitié–Salpêtrière, 47, bd de l’Hôpital, 75651 Paris Cedex 13, France

**Keywords:** Extracorporeal membrane oxygenation, Massive pulmonary embolism, Cardiogenic shock, Long-term quality of life

## Abstract

**Background:**

Despite quick implementation of reperfusion therapies, a few patients with high-risk, acute, massive, pulmonary embolism (PE) remain highly hemodynamically unstable. Others have absolute contraindication to receive reperfusion therapies. Venoarterial-extracorporeal membrane oxygenation (VA-ECMO) might lower their right ventricular overload, improve hemodynamic status, and restore tissue oxygenation.

**Methods:**

ECMO-related complications and 90-day mortality were analyzed for 17 highly unstable, ECMO-treated, massive PE patients admitted to a tertiary-care center (2006–2015). Hospital- discharge survivors were assessed for long-term health-related quality of life. A systematic review of this topic was also conducted.

**Results:**

Seventeen high-risk PE patients [median age 51 (range 18–70) years, Simplified Acute Physiology Score II (SAPS II) 78 (45–95)] were placed on VA-ECMO for 4 (1–12) days. Among 15 (82%) patients with pre-ECMO cardiac arrest, seven (41%) were cannulated during cardiopulmonary resuscitation, and eight (47%) underwent pre-ECMO thrombolysis. Pre-ECMO median blood pressure, pH, and blood lactate were, respectively: 42 (0–106) mmHg, 6.99 (6.54–7.37) and 13 (4–19) mmol/L. Ninety-day survival was 47%. Fifteen (88%) patients suffered in-ICU severe hemorrhages with no impact on survival. Like other ECMO-treated patients, ours reported limitations of all physical domains but preserved mental health 19 (4–69) months post-ICU discharge.

**Conclusions:**

VA-ECMO could be a lifesaving rescue therapy for patients with high-risk, acute, massive PE when thrombolytic therapy fails or the patient is too sick to benefit from surgical thrombectomy. Because heparin-induced clot dissolution and spontaneous fibrinolysis allows ECMO weaning within several days, future studies should investigate whether VA-ECMO should be the sole therapy or completed by additional mechanical clot-removal therapies in this setting.

**Electronic supplementary material:**

The online version of this article (doi:10.1186/s13054-017-1655-8) contains supplementary material, which is available to authorized users.

## Background

Acute, massive high-risk pulmonary embolism (PE) is defined as an embolus sufficiently obstructing pulmonary blood flow to cause right ventricular (RV) failure, hypoxemia, and hemodynamic instability [1]. Although the epidemiology of massive PE is difficult to determine, it remains a significant cause of cardiovascular morbidity and mortality worldwide, with overall in-hospital mortality rates ranging from 25% for patients with cardiogenic shock to 65% for those requiring cardiopulmonary resuscitation [1, 2]. The latest European guidelines enhance the clinical classification, based on the estimated PE-related early mortality risk, defined by in-hospital or 30-day mortality, with high-risk PE being suspected or confirmed in the presence of shock or persistent arterial hypotension [3]. Treatment is based on bedside hemodynamic and respiratory support, unfractionated heparin infusion (UFH), and reperfusion therapy with systemic thrombolytic agents (class IB), surgical pulmonary embolectomy (class IC) or percutaneous catheter-directed thromboaspiration or embolectomy (class IIaC) [3]. Because of contraindications or major clinical instability, a few patients are not amenable to reperfusion therapies or fail to improve after this treatment. For them, venoarterial-extracorporeal membrane oxygenation (VA-ECMO) is one of the most reliable and quickest ways to decrease RV overload, improve RV function and hemodynamic status, and restore tissue oxygenation. Although ECMO is increasingly available and mobile ECMO teams, if locally available, can assure rapid deployment of this salvage therapy, ECMO data in this context are limited [4–7]. We describe herein our tertiary-care center’s experience with VA-ECMO–treated patients with acute, massive, high-risk PE, and report their short- and long-term outcomes.

## Methods

### Patients

We retrospectively analyzed the ECMO database of our 26-bed intensive care unit (ICU), to identify all the patients referred (June 2006–June 2015) with suspected or confirmed high-risk PE, indicating VA-ECMO support. PE was diagnosed using the diagnostic strategy tools of the latest European Society of Cardiology guidelines [3]. VA-ECMO indications were: acute refractory cardiovascular failure, defined as evidence of tissue hypoxia (e.g., extensive skin mottling or elevated blood lactate) concomitant with adequate intravascular volume status; severely diminished RV or left ventricular ejection fraction (RV/LVEF); low cardiac index (≤2.1 L/min/m^2^); sustained hypotension despite high-dose catecholamine infusion (epinephrine ≥1 γ/kg/min or dobutamine ≥20 γ/kg/min + norepinephrine ≥1 γ/kg/min). ECMO exclusion criteria were malignancies with fatal prognosis within 5 years or irreversible neurological pathologies and decisions to limit therapeutic interventions.

VA-ECMO cannulas were surgically inserted by trained cardiovascular surgeons with femoral–femoral 23 F to 29 F–15 F to 18 F cannula as previously described [8–10]. An additional 7 F catheter was systematically inserted into the femoral artery to prevent leg ischemia. For highly unstable patients, our institution’s Mobile ECMO Unit traveled to primary-care hospitals with a portable ECMO system, implanted the device at beside in the ICU and transported the patient to our center. When cannulation was done during surgical pulmonary embolectomy, the inflow cannula was placed in the right atrium (RA) and the outflow line in the main pulmonary artery (PA). Pump speed was adjusted to obtain blood flow of 2.5–3.5 L/min with intravenous UHF administered to maintain the activated partial thromboplastin time at two to three times control levels (see Additional file 1 for details on ECMO management)..

### Pre-ECMO data collection

At ICU admission, we collected the following information: demographics (age, sex, body mass index), admission disease-severity scores (severity of underlying conditions according to the McCabe and Jackson [11] and Charlson scores [12]); presence of venous-thromboembolic risk factors; Simplified Acute Physiology Score (SAPS) II [13] and Sequential Organ Failure Assessment (SOFA) score [14]). During the pre-ECMO period, the inotrope score, defined as dobutamine dose (γ/kg/min) + [norepinephrine dose (γ/kg/min) + epinephrine dose (γ/kg/min)] × 100 [15]; cardiac arrest with its related “no-flow” and “low-flow” durations; blood gas analyses, and troponin Ic were noted. The following Doppler echocardiography variables were always recorded: RV/LV dimension ratio, LVEF, and visualization of a PA thrombus, before ECMO insertion. Similarly, proximal PE and pulmonary infarction on chest computed tomography (CT) scans were recorded. Lastly, pre-ECMO reperfusion therapies (including thrombolysis, surgical thrombectomy or percutaneous catheter-directed thromboaspiration or embolectomy), ECMO-related complications, and post-ECMO information were collected.

### Outcome variables

The main outcome variables included ECMO weaning, survival to hospital discharge, 90-day survival, and long-term survival (evaluated in September 2015). We also calculated each patient’s SOFA scores at cannulation and days 1, 3, and 7; the inotrope score 24 hours post-cannulation; ECMO and mechanical ventilation durations. Lastly, in-ICU complications, e.g., severe hemorrhage, arterial ischemia, surgical wound infection, stroke, and renal replacement therapy requirement, were recorded. Bleeding complications were reported using the Global Utilization of Streptokinase and TPA for Occluded arteries (GUSTO) classification [16, 17]. Briefly, severe life-threatening hemorrhage was intracerebral bleeding or resulted in substantial hemodynamic compromise requiring treatment (GUSTO 1). GUSTO 2 defined moderate bleeding as the need for transfusion, whereas GUSTO 3 referred to other bleeding, not requiring transfusion or causing hemodynamic compromise. Lastly, the number of packs of blood products transfused was also collected.

In September 2015, a telephone interview with survivors evaluated their health-related quality of life (HRQOL), using the French version of the Medical Outcome Study-Short Form 36-item (SF-36) questionnaire. Its 36 items are combined to evaluate eight domains (physical functioning, role-physical, body pain, general health, vitality, social functioning, role-emotional, and mental health) [18]. The aggregate physical (PCS) and mental component summary scores (MCS) were then computed as recommended [18]. Our patients’ mean SF-36 levels were compared to those obtained for French age- and sex-matched controls with no adverse conditions. Anxiety and depression symptoms were assessed with the Hospital Anxiety and Depression Scale [19], with respective HAD-A and HAD-D subscale scores ≥8/21 considered clinically significant [19]. Post-traumatic stress disorder (PTSD)-related symptoms were assessed with the Impact of Event Scale (IES) [20], with total IES scores ≥30/75 points indicating a high risk for PTSD. Other long-term outcome variables were: instrumental activities of daily living (IADL) scale score [21], recurrence of thromboembolic events, chronic dyspnea, chronic thromboembolic pulmonary hypertension (CTEPH) diagnosis, persistent anticoagulant use, and return to work. To put our massive PE ECMO-treated patients’ questionnaire scores into perspective, we searched the literature for other studies reporting long-term outcomes of ECMO-treated patients, e.g., refractory septic shock [22] or acute respiratory disease syndrome (ARDS) [23].

In accordance with the ethical standards of our hospital’s Institutional Review Board and French law, informed consent was not necessary for analyses of demographic, physiological, and hospital-outcome data, because this retrospective observational study did not modify existing diagnostic or therapeutic strategies. The National Commission for Informatics and Liberties (CNIL) approved this study (number 1950673). Survivors gave oral consent to participate in the telephone interview conducted by the same investigator (MS).

### Literature review

We conducted a systematic MEDLINE database literature review through the PubMed search engine with a global search strategy applying pre-specified selection and outcome. We combined the terms “extracorporeal life support” or “extracorporeal membrane oxygenation” with the terms “pulmonary embolism” or “acute pulmonary embolism” or “massive pulmonary embolism”. See Additional file 1 for details on methodology and review results.

### Statistical analyses

Results are expressed as numbers (%) or median (range). Continuous variables were compared with Student’s *t* test or the Mann–Whitney *U* test, as appropriate, whereas categorical variables were compared with chi-square tests. Analyses were performed using StatView v5.0 software (SAS Institute Inc., Cary, NC, USA) and a two-sided *p* < 0.05 defined significance.

## Results

### Study population

**Fig. 1:**
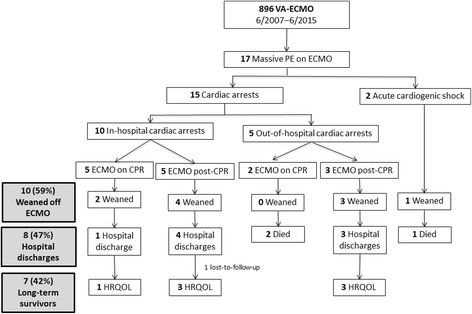
Study flow chart. *CPR* cardiopulmonary resuscitation, *HRQOL* health- related quality of life, *PE* pulmonary embolism, *VA-ECMO* venoarterial-extracorporeal membrane oxygenation

During the 10-year study period, 17 patients [11 females; median age 51 (18–70) years] received ECMO for suspected (*n* = 2) or confirmed (*n* = 15) massive PE (Fig. 1). Median SAPS II and SOFA score were high, respectively, 78 (45–95) and 12 (8–16); 11 (65%) patients had predisposing factors for venous thromboembolism (Table 1). Fifteen (82%) patients suffered pre-ECMO cardiac arrest, with seven (41%) of them cannulated during cardiopulmonary resuscitation. Two other patients received ECMO for refractory cardiogenic shock. Our institution’s Mobile ECMO Unit implanted peripheral femoral–femoral VA-ECMO into seven (41%) patients; nine (53%) were cannulated at beside in our department. Two patients received ECMO for hemodynamic status deterioration during surgical thrombectomy for acute pulmonary embolism, including one with a RA–PA central configuration. All patients required hemodynamic support with vasoactive drugs, resulting in a median inotrope score of 100 (1.8–760) μg/kg/min at ECMO cannulation. Pre-ECMO median blood pressure, pH and blood lactate were, respectively: 42 (0–106) mmHg, 6.99 (6.54–7.37) and 13 (4–19) mmol/L. Transthoracic or transesophageal echocardiography visualized major RV dilation in all patients (RV/LV dimensions ratio 1.3 [0.7–1.6]), with a proximal PA thrombus in three. Chest CT scan confirmed PE in 12 patients of whom ten had proximal PE. Eight patients received unsuccessful systemic fibrinolytic therapy pre-ECMO according to standard protocols [24]. Peripheral VA-ECMO was implanted for refractory cardiogenic shock several hours post-surgical embolectomy for one patient, whereas another was cannulated during the procedure in the operating room (RA–PA central configuration). Two patients underwent catheter-directed thromboaspiration: one on ECMO and the other failed pre-ECMO because of hemodynamic instability. As described in Tables 1 and 2, 24 hours on ECMO rapidly corrected pH (6.99 [6.54–7.37] vs. 7.42 [7.19–7.69]) and serum lactate (13.3 [4.2–19.0] vs. 3.2 [1.1–12.3] mmol/L). In addition, one patient’s follow-up CT scans showed major clot dissolution with residual thrombi 15 days later, including 8 days on ECMO and prolonged heparin treatment but without systemic fibrinolytic therapy (see Additional file 2).

**Table 1:** Clinical characteristics of the 17 patients at the time of VA-ECMO implantation

Variable	Value
Age, yr, median (range)	51 (18–70)
Male	6 (35)
Body mass index, kg/m^2^	29 (20–35)
McCabe and Jackson score ≥2	3 (18)
Charlson score ≥2	5 (29)
SAPS II	78 (45–95)
SOFA score	12 (8–16)
Predisposing factors for venous thromboembolism	11 (65)
Postoperative status	4
Immobility due to sitting	2
Oral contraception	1
Previous venous thromboembolism	3
Postpartum period	3
Hospitalization for HF or AF (within previous 3 months)	2
ECMO implantation by Mobile ECMO Unit	7 (41)
Femoral–femoral VA-ECMO	16 (94)
Pre-ECMO cardiac arrest	15 (88)
No-flow time, min	0 (0–11)
Low-flow time, min	30 (10–85)
ECMO during cardiopulmonary resuscitation	7 (41)
Pre-ECMO systolic blood pressure, mmHg	55 (0–130)
Pre-ECMO mean blood pressure, mmHg	42 (0–106)
Pre-ECMO heart rate, bpm	95 (0–177)
Pre-ECMO inotrope score, μg/kg/min	100 (1.8–760)
pH	6.99 (6.54–7.37)
Blood lactate, mmol/L	13.3 (4.2–19.0)
Bicarbonate, mmol/L	12 (3–25)
Troponin I, μg/mL	2.2 (0.1–23.7)
PaO_2_/FiO_2_ ratio	198 (32–674)
PaCO_2_, mmHg	48 (17–102)
Bilirubin, mmol/L	8 (6–124)
Prothrombin activity, %	34 (10–72)
Pre-ECMO cardiac echocardiography	17 (100)
RV dilation	17 (100)
Pulmonary artery thrombus	3 (18)
LVEF, %	40 (5–60)
Pre-ECMO chest CT scan	12 (71)
Proximal PE	10 (59)
Pulmonary infarction	3 (18)
RV/LV dimensions ratio	1.3 (0.7–1.6)
Pre-ECMO systemic fibrinolytic therapy	8 (47)
Pre-ECMO surgical thrombectomy	2 (12)
Pre-ECMO catheter-directed thromboaspiration	1 (6)

Categorical variables are expressed as *n* (%) and continuous variables as median (range), unless stated otherwise

*VA-ECMO* venoarterial-extracorporeal membrane oxygenation, *SAPS II* Simplified Acute Physiology Score II, *SOFA* Sequential Organ Failure Assessment, *AF* atrial fibrillation, *HF* heart failure, *RV* right ventricular, *LVEF* left ventricular ejection fraction

**Table 2:** ICU events and outcomes of ECMO-treated massive PE patients according to 90-day survival status

Event/outcome	All patients (*n* = 17)	Non-survivors (*n* = 9)	Survivors (*n* = 8)	*p*
SAPS II at ICU admission	78 (45–95)	83 (71–95)	58 (45–91)	0.04
Shock onset-to-ECMO interval, h	3 (1–24)	3 (1–24)	3.5 (1–12)	0.92
Extracorporeal blood flow during the 1st 24 h, L/min	3.3 (3.0–4.2)	3.3 (3.2–4.2)	3.2 (3.0–3.9)	0.19
SOFA score at ECMO cannulation	12 (8–19)	15 (11–19)	12 (8–15)	0.11
Inotrope score at ECMO cannulation, μg/kg/min	100 (2–760)	75 (2–730)	143 (92–760)	0.17
Inotrope score after 24 h of ECMO, μg/kg/min	50 (0–660)	75 (41–660)	6 (0–51)	0.001
pH ECMO-day 1	7.42 (7.19–7.69)	7.40 (7.19–7.57)^a^	7.44 (7.32–7.69)	0.79
Blood lactate ECMO-day 1, mmol/L	3.2 (1.1–12.3)	4.5 (1.1–12.3)^a^	2.3 (1.1–3.5)	0.17
SOFA score				
ECMO-day 1	14 (11–18)	14 (13–18)	12 (11–16)	0.04
ECMO-day 3	13 (8–18)	15 (12–18)	13 (8–15)	0.03
ECMO-day 7	6 (1–19)	10 (7–13)	6 (1–19)	0.29
In-ICU complications				
Hemorrhage ≤ GUSTO 2	15 (88)	8 (89)	7 (88)	0.92
RRT	13 (76)	6 (67)	7 (88)	0.31
Stroke	4 (24)	1 (11)	3 (38)	0.2
Surgical wound infection	2 (12)	0	2 (25)	–
Arterial ischemia	1 (6)	0	1 (12)	–
Packed red-cell units transfused	4 (0–29)	4 (0–6)	9 (0–29)	0.7
Fresh-frozen plasma units transfused	5 (0–11)	3 (0–8)	8 (0–11)	0.7
Tracheotomy	2 (12)	0	2 (25)	–
ECMO duration, days	4 (1–12)	3 (1–13)	4 (3–11)	0.28
MV duration, days	10 (1–43)	3 (1–24)	13 (1–43)	0.03
ICU LOS, days	10 (1–91)	3 (1–24)	17 (7–91)	0.009
Hospital LOS, days	22 (1–135)	6 (1–36)	45 (22–135)	0.004

Categorical variables are expressed as n (%) and continuous variables as median (range)

*ICU* intensive care unit*, ECMO* extracorporeal membrane oxygenation, *PE* pulmonary embolism, *SAPS II* Simplified Acute Physiology Score II, *SOFA* Sequential Organ Failure Assessment, *GUSTO* Global Utilization of Streptokinase and TPA for Occluded arteries, *RRT* renal replacement therapy, *MV* mechanical ventilation, *LOS* length of stay

^a^Three patients died within less than 24 h on ECMO

### 90-Day survival and hospital discharge

Table 2 reports ECMO-related complications and short-term outcomes according to 90-day status. Almost all patients experienced at least one major ECMO-related complication; 15 (88%) had a moderate-to-severe hemorrhage classified as GUSTO ≤2 with a median of 4 (0–29) packed red-cell and 5 (0–11) fresh-frozen plasma units transfused. Eight out of 15 patients (47%) had a SOFA_liver_ ≥ 1 before ECMO cannulation, which was not associated with a higher rate of severe bleeding (*p* = 0.94).

Thirteen (76%) patients received renal replacement therapy during their ICU stay. Both complications had no impact on 90-day survival. Other complications included: ischemic stroke in four patients, with two recovering normal neurological function at hospital discharge; arterial ischemia in two patients, one each underwent lower limb or toe amputation, and two surgical cannula-related, wound-infection debridement.

**Fig. 2:**
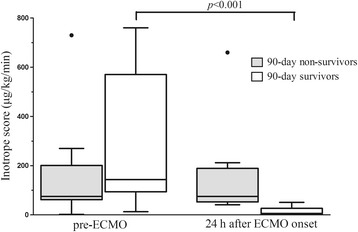
Box plots of the inotrope score change between pre- and post-VA-ECMO cannulation according to patients’ 90-day status. *Bold horizontal lines* are medians; *lower and upper box limits* are 25th–75th percentiles; *T-bars* represent 10th–90th percentiles. *ECMO* extracorporeal membrane oxygenation

Nine patients died within 90 days post-ICU admission: seven on ECMO (six of cardiac arrest-related multiorgan failure and one of refractory cardiogenic shock despite ECMO support), and one each after successful ECMO weaning of secondary cardiogenic shock with multiorgan failure or PE recurrence with sudden death 6 days post-ECMO removal despite adequate anticoagulation. One patient, still hospitalized 90 days post-ICU admission, was discharged after 135 days in the hospital. It is worth noting that only one out of the seven patients cannulated while undergoing cardiopulmonary resuscitation (CPR) was discharged alive from hospital (Fig. 1). The eight (47%) hospital survivors, including the patient with central ECMO, were discharged after 4 (3–11) days on ECMO, 17 (7–91) in-ICU days and 45 (22–135) in-hospital days. Compared with patients who died within 90 days, it is worth noting that 90-day survivors had significantly lower inotrope scores 24 hours post-cannulation (Fig. 2), and lower SAPS II and SOFA scores on ECMO days 1 and 3.

### Long-term outcomes

**Fig. 3:**
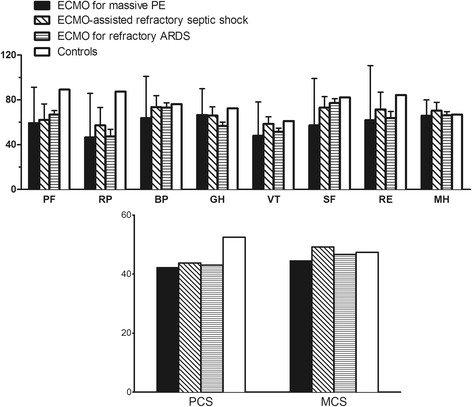
Comparison of median Short Form-36 scores of our high-risk massive PE survivors treated with VA-ECMO after median 19-month follow-up post-hospital discharge and their age- and sex-matched control subjects [18], 67 venovenous-ECMO-treated acute respiratory distress syndrome (ARDS) survivors at 17-month follow-up [23] and ten VA-ECMO-treated septic shock patients [23]. Higher scores denote better health-related quality of life. ECMO extracorporeal membrane oxygenation, *ARDS* acute respiratory disease syndrome, *PF* physical functioning, *RP* role-physical, *BP* body pain, *GH* general health, *VT* vitality, *SF* social functioning, *RE* role-emotional, *MH* mental health, *PCS* physical component score, *MCS* mental component score

IADL, SF-36, HAD, and IES questionnaires were administered to seven of the eight long-term survivors after median follow-up of 19 (4–74) months post-hospital discharge. One patient, known to be alive at home 1 month before follow-up, could not be reached. Daily-living activities were normal for five patients, whereas two others reported moderate limitations due to physical impairment after lower limb or toe amputation: IADL scores of 24 and 20, respectively. Comparison with age- and sex-matched controls highlighted limitations of all physical domains but preserved mental health function. However, PCS and MCS were similar to those of other ECMO-assisted refractory septic shock and ECMO-treated refractory ARDS patients (Fig. 3). Our respondents exhibited significant anxiety (28%) or depression symptoms (43%), or were at risk for PTSD (28%), with only two (28%) returning to their previous work. Eighty-six percent of long-term survivors were still taking anticoagulants; none reported chronic dyspnea, PE recurrence or CTEPH diagnosis during their medical follow-up.

## Discussion

To our knowledge, this is the largest follow-up study on VA-ECMO-treated life-threatening PE in the modern era. Despite extreme disease severity at ECMO implantation, multiorgan failure and 91% SAPS II-predicted mortality, 47% of these patients were alive at 90 days with acceptable long-term HRQOL. Nevertheless, after median 19-month follow-up, physical limitations were frequently reported, with normal mental health function. In addition, anxiety, depression or PTSD symptoms persisted for almost one-third of the survivors.

To date, literature on VA-ECMO, as rescue treatment for extremely severe, massive PE, had been limited to small case series or case reports with no long-term outcome evaluation see Additional file 3 (Table 3). In 2007, Maggio et al. reported on 21 cohort patients diagnosed with high-risk PE between 1992 and 2005 [4]: 19 were cannulated with VA bypass, six received pre-ECMO thrombolytic therapy and eight were cannulated after suction or surgical pulmonary embolectomy failed. Overall survival was 62%, with catastrophic neurological events responsible for 50% of the deaths. In our study, severe bleeding episodes occurred in 15 (88%) patients requiring packed red-cell and/or fresh-frozen plasma transfusions. Major bleeding, including intracranial hemorrhage is a well-recognized ECMO complication [25], with numerous identified risk factors, e.g., thrombocytopenia, vasopressor requirement, and cardiopulmonary resuscitation [25, 26]. Due to previous thrombolytic treatment and curative anticoagulation, life-threatening PE on ECMO may carry additional risk factors of major bleeding during this circulatory support.

**Table 3:** Studies on patients with acute, massive, high-risk PE on VA-ECMO support included in the systematic review

Reference	Inclusion dates	Patients, *n*	Pre-ECMO		Fibrinolytic therapy (%)	Mechanical PE removal on ECMO (*n* patients)	VA-ECMO-related complications (*n* patients)	Survival (%)
			Cardiac arrest (%)	Mechanical PE removal (*n* patients)				
Kawahito et al. [35]	1994–1998	7	71	0	100	3 surgical pulmonary embolectomies	0	57
Maggio et al. [4]	1992–2005	21^a^	38	6 suction and 2 surgical pulmonary embolectomies	29	1 suction and 2 surgical pulmonary embolectomies	4 catastrophic neurological events; 1 dislodged arterial cannula	62
Sakuma et al. [36]	1983–2006	7	NR	0	86	1 suction and 1 surgical pulmonary embolectomies	NR	57
Malekan et al. [5]	2005–2011	4	NR	0	0	1 suction pulmonary embolectomy	None	100
Munakata et al. [30]	1992–2008	10	90	2 suction pulmonary embolectomies	100	7 suction pulmonary embolectomies	2 major bleeding	70
Omar et al. [37]	2007–2011	4	50	1 suction and 2 surgical pulmonary embolectomies	25	None	NR	25
Maj et al. [38]	NR	6	100	None	66	1 surgical pulmonary embolectomy	3 major bleeding	33
Swol et al. [39]	2008–2014	5	100	None	60	1 surgical pulmonary embolectomy	1 major bleeding	40
Cho et al. [40]	2000–2013	13	NR	None	15	11 surgical pulmonary embolectomies	NR	NR
This study	2006–2015	17	88	1 suction and 1 surgical pulmonary embolectomies	47	1 suction and 1 surgical pulmonary embolectomies	15 major bleeding	47^b^

*PE* pulmonary embolism, *VA-ECMO* venoarterial-extracorporeal membrane oxygenation, *NR* not reported

^a^Nineteen of the 21 patients were cannulated for VA-ECMO and two were placed on venovenous-ECMO

^b^Reported at 90 days

Our results highlight that ECMO can provide lifesaving hemodynamic support at bedside for critically ill patients too unstable to tolerate other interventions or refractory to other therapies. A recent survival-prediction model indicated a lower predicted chance of survival for each associated extracardiac organ failure at ECMO onset, which starkly illustrates the crucial impact of VA-ECMO timing for refractory cardiogenic shock [27]. To shorten this interval, mobile ECMO teams able to implant a portable and quick-to-prime ECMO circuit just after the emergency call [28] might help clinicians overcome these difficulties.

Current guidelines for high-risk PE advocate using reperfusion therapy with systemic thrombolytic agents or surgical pulmonary embolectomy [3]. However, those recommendations might be questionable for the sickest patients in severe shock or cardiac arrest, when thrombolysis takes time to be effective and surgery is not immediately available. Therefore, VA-ECMO could be used to rescue patients when thrombolytic treatments fail or as temporary hemodynamic support prior to surgical [29] or catheter-based embolectomy [30]. However, surgical embolectomy is a major intervention requiring sternotomy and cardiopulmonary bypass that carries significant morbidity and mortality in this context of advanced shock and multiorgan failure; hence, VA-ECMO might also be used alone until heparin-induced and spontaneous endogenous thrombolysis permit weaning-off support [5]. Herein, sufficient clot dissolution allowing ECMO removal was obtained within 4 (3–11) days for the eight patients rescued by VA-ECMO alone.

The other rationale supporting surgical thrombectomy on ECMO is to limit the CTEPH risk [29], which has been reported to be 0.1–9.1% for patients within the first 2 years after symptomatic PE [31]. However, data confirming that hypothesis are lacking. Notably, none of our long-term survivors developed CTEPH. In addition, despite significant mechanical PA obstruction by massive PE, thrombectomy to prevent CTEPH is not yet systematically advocated [3]. The lack of linear correspondence between the degree of mechanical obstruction and CTEPH risk, because of concomitant small-vessel pulmonary arteriopathy [32], makes the benefit of adding surgical thrombectomy in this context questionable. Lastly, a recent systematic review of case reports and case series published over the past 20 years found similar outcomes for patients who underwent surgical or catheter embolectomy or no additional therapies on ECMO [7]. The benefit of mechanical removal therapies, e.g., catheter or surgical thrombectomy, over exclusive VA-ECMO use warrants further investigation.

Despite very severe disease at ECMO initiation, the 47% 90-day survival observed for our series is comparable with results reported in studies included in our systematic review (Table 3) and with the 42% hospital-survival rate of a large international cohort of ECMO-treated refractory cardiogenic shock patients [27]. Despite high numbers of our patients with pre-ECMO cardiac arrest or cannulated during cardiopulmonary resuscitation, our survivors’ survival rate was also higher than those reported for ECMO-treated in- and out-of-hospital cardiac arrest (28.8% and 4%, respectively) [33, 34]. However, HRQOL evaluated after median 19-month follow-up, was still impaired, compared to sex- and age-matched controls, especially concerning SF-36 physical health and social-functioning domains, while general and mental health were considered satisfactory. Although, case-mix differences make comparisons between series difficult, we observed than our extremely ill patients’ SF-36 scores were similar to those of both ECMO-assisted refractory shock [22] and ECMO-treated refractory ARDS patients [23]. Nevertheless, the burden of ECMO-induced physical limitations for our ECMO-treated survivors was still perceptible 19 months post-hospital discharge with back-to-work impact. Although thoroughly described in previous case series [8–10], ECMO-related long-term physical sequelae have not been investigated. Future studies are warranted to prevent these complications and improve their long-term management.

Our study’s strengths are the larger number of consecutive patients included and their detailed characterization, and its longitudinal design with median survivor follow-up 19 months post-ICU discharge. However, our study also has limitations. First, it is a retrospective, single-center study. Second, the self-assessed persistently impaired physical health and vitality might not be specific to PE but may represent sequelae of any severe disease requiring prolonged ICU stay and ECMO, including critical illness, muscle wasting, and weakness. Third, we did not perform protocolized follow-up based on long-term cardiac echocardiography and imaging to detect CTEPH development. Further studies focusing on this point are needed to support long-term safety of an ECMO strategy without additional mechanical clot-removal therapies. Lastly, PE diagnosis was confirmed in 15 out of the 17 patients. The remaining two patients had high massive PE suspicion but died within 24 hours after ICU admission without chest CT scan performed. The family refused autopsy. However, they both had prolonged cardiac arrest with massive RV dilatation on cardiac echocardiography, predisposing factors for venous thromboembolism, and no evidence of right myocardial infarction.

## Conclusions

In conclusion, long-term survival of our 17 VA-ECMO–treated patients with life-threatening, massive PE reached 47%. Although only limited data support VA-ECMO effectiveness in this context at present, our results suggest that it could be a lifesaving rescue therapy to rapidly restore hemodynamic status when thrombolytic therapy fails or when the patient is deemed too sick to benefit from medical or surgical treatments. Considering that heparin-induced clot dissolution and spontaneous fibrinolysis allows ECMO weaning after only a few days on support, the benefit of additional mechanical clot-removal therapies, e.g., catheter-based or surgical thrombectomy on ECMO, also warrant investigation.

## Additional files

Additional file 1: Supplementary methods (DOCX 18 kb)

Additional file 2: (A) Computed tomography (CT) scan showing a saddle embolus extending into the left and right pulmonary arteries. (B) The same patient’s follow-up CT scan obtained 9 days later on VA-ECMO. (C) CT scan obtained 15 days after ICU admission with successful ECMO weaning after 10 days on circulatory support. (DOCX 462 kb)

Additional file 3: Flow diagram illustrating the identification, selection, and exclusion of articles used in the review. (DOCX 95 kb)

## Abbreviations

ARDS: Acute respiratory disease syndrome
CT: Computed tomography
CTEPH: Chronic thromboembolic pulmonary hypertension
GUSTO: Global Utilization of Streptokinase and TPA for Occluded arteries
HAD-A and HAD-D: Hospital Anxiety and Depression Scale
HRQOL: Health-related quality of life
IADL: Instrumental activities of daily living
ICU: Intensive care unit
IES: Impact of Event Scale
LVEF: Left ventricular ejection fraction
MCS: Mental component summary scores
PA: Pulmonary artery
PCS: Physical component summary scores
PE: Pulmonary embolism
PTSD: Post-traumatic stress disorder
RA: Right atrium
RV: Right ventricular
SAPS II: Simplified Acute Physiology Score II
SF-36: Short Form 36-item Questionnaire
SOFA: Sequential Organ Failure Assessment
UFH: Unfractionated-heparin infusion
VA-ECMO: Venoarterial-extracorporeal membrane oxygenation
