# Supplementary material for: Life-threatening massive pulmonary embolism rescued by venoarterial-extracorporeal membrane oxygenation
Source: Crit Care. 2017 Mar 28;21:76. doi: 10.1186/s13054-017-1655-8 (PMC5369216; doi:10.1186/s13054-017-1655-8)
Supplement: Supplementary file 1 — Supplementary methods (DOCX 18 kb) [file 13054_2017_1655_MOESM1_ESM.docx]

**Additional file 1 Supplementary Methods**

**Patient management on ECMO for acute, massive PE**

Pump speed was adjusted to obtain blood flow of 2.5–3.5 L/min with intravenous UHF administered to maintain the activated partial thromboplastin time at 2–3-times control levels. An ECMO-weaning trial was attempted for hemodynamically stable patients who underwent ECMO flow reduction trials to <1.5 L/min under clinical and Doppler echocardiography monitoring, as previously described [8]. The membrane and ECMO circuit were changed in the following situations: defective oxygenation or CO2 elimination by the membrane, device-associated massive intravascular hemolysis, circuit-linked severe thrombopenia or hypofibrinogenemia. The hemoglobin threshold for red-cell transfusion was 7–8 g/dL and platelet transfusion was discouraged except for severe thrombopenia (<20 G/L) or when thrombopenia was accompanied by bleeding. No antibiotic prophylaxis was administered at peripheral ECMO implantation or during support, (only the patient who was cannulated during surgical embolectomy received prophylactic antibiotics).

**Literature review**

We conducted a systematic MEDLINE-database literature review through the PubMed search engine with a global search strategy applying prespecified selection and outcome criteria. We combined the terms ‘extracorporeal life support’ or ‘extracorporeal membrane oxygenation’ and ‘pulmonary embolism’ or ‘acute pulmonary embolism’ or ‘massive pulmonary embolism’ using the Boolean operator ‘AND’. Language was limited to English-language articles. We also searched the references of identified studies. Randomized–controlled trials, observational studies and case series reporting on adult patients with pulmonary embolism rescued by VA-ECMO with their therapeutic management, ECMO-related complications and outcomes were eligible. Studies on children or newborns and case series with fewer than two cases were excluded.

Two authors (M.S. and N.B.) independently reviewed the retrieved abstracts and assessed eligibility. A third author (G.H.) determined eligibility in the case of disagreement. The following information was extracted: dates of inclusions, numbers of patients, pre-ECMO features (cardiac arrest and mechanical clot-removal therapies), fibrinolysis use, on-ECMO characteristics (mechanical clot-removal therapies, ECMO-related complications) and hospital or ICU-survival.
