# Supplementary material for: Life-threatening massive pulmonary embolism rescued by venoarterial-extracorporeal membrane oxygenation
Source: Crit Care. 2017 Mar 28;21:76. doi: 10.1186/s13054-017-1655-8 (PMC5369216; doi:10.1186/s13054-017-1655-8)
Supplement: Supplementary file 2 — (A) Computed tomography (CT) scan showing a saddle embolus extending into the left and right pulmonary arteries. (B) The same patient’s follow-up CT scan obtained 9 days later on VA-ECMO. (C) CT scan obtained 15 days after ICU admission with successful ECMO weaning after 10 days on circulatory support. (DOCX 462 kb) [file 13054_2017_1655_MOESM2_ESM.docx]

**Additional file 2.** (A) Computed-tomography (CT) scan showing a saddle embolus extending into the left and right pulmonary arteries. (B) The same patient’s follow-up CT scan obtained 9 days later on VA-ECMO. (C) CT scan obtained 15 days after ICU admission with successful ECMO-weaning after 10 days on circulatory support.

**
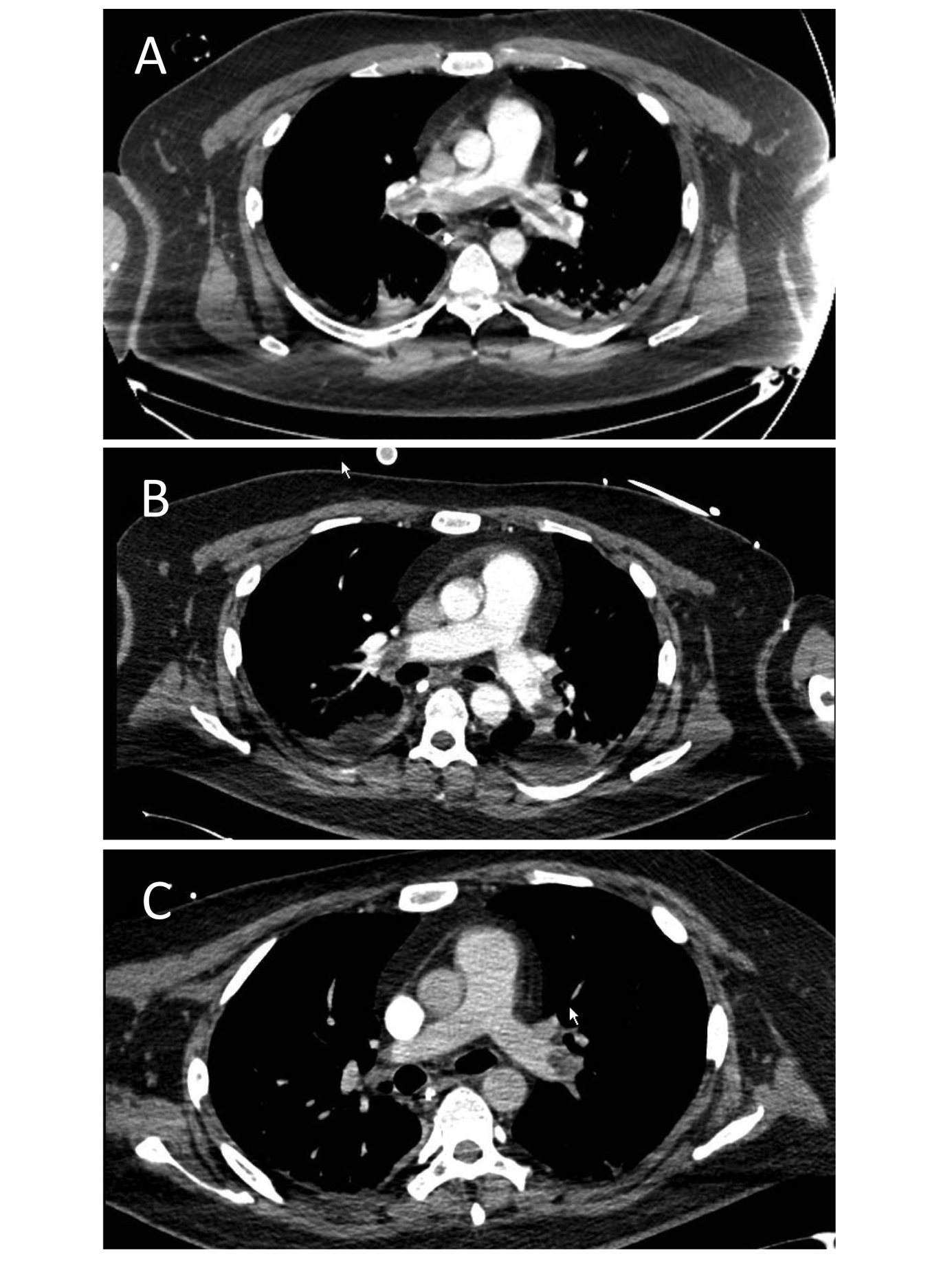
**
