# Supplementary material for: Life-threatening massive pulmonary embolism rescued by venoarterial-extracorporeal membrane oxygenation
Source: Crit Care. 2017 Mar 28;21:76. doi: 10.1186/s13054-017-1655-8 (PMC5369216; doi:10.1186/s13054-017-1655-8)
Supplement: Supplementary file 3 — Flow diagram illustrating the identification, selection, and exclusion of articles used in the review. (DOCX 95 kb) [file 13054_2017_1655_MOESM3_ESM.docx]

**Additional file 3.** Flow diagram illustrating the identification, selection and exclusion of articles used in the review

**
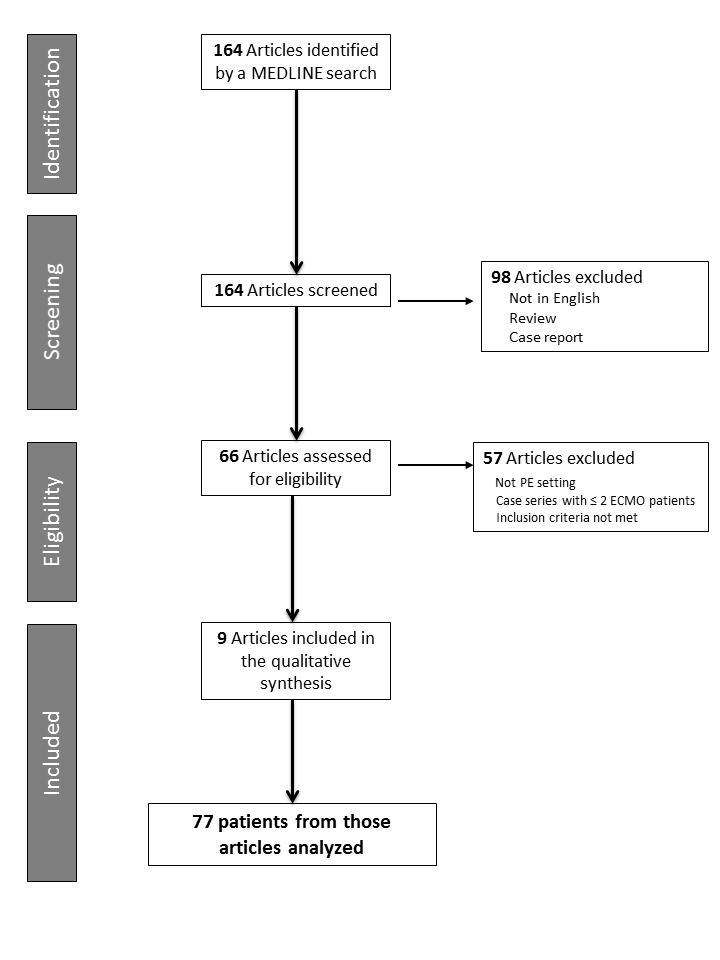
**
